# The impact of supervisor control on employee performance and wellbeing in COVID-19 telecommuting: based on the Job Demands-Resources model

**DOI:** 10.3389/fpsyg.2026.1789163

**Published:** 2026-05-25

**Authors:** Jinhua Gan, Xiangping Zhan, Lei Yan, Chen Sun, Linchuan Yang, Yujia Wei

**Affiliations:** 1 Department of Psychology, College of Education and Sports Sciences, Yangtze University, Jingzhou, China; 2 Shandong Judicial Police Vocational College, Jinan, China; 3 Social Psychology Research Center, Yangtze University, Jingzhou, China; 4 School of Psychology, Central China Normal University, Wuhan, China; 5 Key Laboratory of Adolescent Cyberpsychology and Behavior (CCNU), Ministry of Education, Wuhan, China; 6 College of Economics & Management, Three Gorges University, Yichang, China

**Keywords:** COVID-19 telecommuting, supervisor control, supervisor support, ego depletion, the Job Demands–Resources (JD-R) model

## Abstract

**Background:**

Since the COVID-19 pandemic, telecommuting has become a common work arrangement, highlighting the critical role of supervisors in influencing employee performance and wellbeing. However, research on how supervisor behavior affects employees in this context remains limited. This study examines how supervisor control impacts employee task performance and sleep quality during COVID-19 telecommuting, using the Job Demands-Resources (JD-R) model as a theoretical framework. It also explores the moderating roles of supervisor and family support.

**Methods:**

Data were collected from 303 Chinese employees during the nationwide lockdown, with two assessments spaced 2 weeks apart.

**Results:**

The results indicate that ego depletion mediates the relationship between supervisor control and both task performance and sleep problems. Supervisor support moderates this relationship by buffering the negative effects of supervisor control on ego depletion, while family support does not have a buffering effect.

**Conclusion:**

This study provides empirical support for the JD-R model in the context of telecommuting during the pandemic, and underscores the importance of supervisor support in mitigating the adverse effects of high control environments.

## 1 Introduction

The COVID-19 pandemic has witnessed widespread adoption of telecommuting and introduced new forms of surveillance when working from home. This shift occurs as supervisors face challenges in monitoring employee performance (Kniffin et al., 2021). Supervisor control, as a means to enhance organizational performance, involves employees' perception of supervisors monitoring their performance and progress using various online tools, including requesting daily reports, clocking in and out through computer applications, and attending online meetings (Gan et al., 2022).

This concept is distinct from abusive supervision or reduced autonomy, as it emphasizes the neutral, task-focused managerial behavior of supervisors monitoring work processes in remote work. Excessive supervisor control may lead to reduced autonomy, yet it has no inherent association with abusive supervision. In the context of telecommuting, prior research predominantly emphasized the positive impact of supervisor support (Chong et al., 2020); however, the influence of supervisor control received less attention and has not yet reached a consensus. For organizations, supervisor control is regarded as an effective means to enhance organizational performance and productivity (Allen et al., 2015); while for employees, it offers opportunities for immediate feedback on their performance. Yet, excessive and frequent control can lead to increased perceived stress and privacy invasion (Kniffin et al., 2021). For instance, Wang et al. (2021) discovered that monitoring, as a virtual work characteristic, was viewed by employees as a tool to enhance task focus and combat procrastination during the pandemic telecommuting. However, their subsequent survey only revealed the adverse effects of monitoring, notably leading to work-family interruptions and reduced wellbeing. Furthermore, while prior research has linked supervisor control during COVID-19 telecommuting to employee work and health outcomes (Gan et al., 2022), it has not sufficiently unpacked the underlying psychological pathways through which this relationship operates. This leaves a critical gap in understanding the full impacts of supervisor control in pandemic-era telework settings.

The Job Demands-Resources (JD-R) model provides a useful framework for elucidating the impact of job characteristics on performance and wellbeing in the context of COVID-19 telecommuting (Bilotta et al., 2021). According to the JD-R model (Bakker and Demerouti, 2016), job characteristics can be categorized as either job demands, which trigger the health impairment process, or job resources, which activate the motivational process. Within the impairment process, elevated job demands may induce strain, subsequently contributing to health issues. Conversely, job resources can mitigate the adverse effects of job demands on strain (Bakker et al., 2005). Empirical studies have supported the roles of job demands and resources in shaping health impairment and motivational processes (e.g., Ratanachina et al., 2026). Rudolph et al. (2022) suggested that distinct leader behaviors may be interpreted by employees as stressors or resources, and supervisors may simultaneously play roles related to demands and resources. Supervisor control can be considered a demand, as research indicates that employees subjected to intensive monitoring by their superiors are more prone to burnout (e.g., Hetland et al., 2007; Stordeur et al., 2001). Meanwhile, supervisor support can be regarded as a resource; for example, organizational support for telecommuting tasks empowers employees by increasing their perceived control over their work environment, subsequently enhancing their coping efficacy (Richardson et al., 2008). Therefore, by introducing the JD-R model to investigate the mechanism and boundary condition of supervisor control on employee performance and wellbeing, this study may advance the knowledge of how supervisor control affects employees' outcomes, and the conditions shaping these associations.

The present study contributes to the existing literature in the following three ways. Firstly, achieving high performance as well as maintaining wellbeing among remote workers is indicative of successful telecommuting (Gohoungodji et al., 2022); while leaders play a crucial role in either constraining or supporting employees in their work (Wang et al., 2021). Establishing the link between supervisor control and employee outcomes is imperative for guiding supervisors in adopting appropriate behaviors to effectively manage and support telecommuters during times of crisis. Secondly, grounded in the JD-R model, we have explored the mechanisms through which supervisor control influences critical work-related and wellbeing outcomes for employees. While research on supervisor behavior in the context of telecommuting has emerged in recent years (Gan et al., 2022), the specific impact of supervisor behavior on employees has not been thoroughly examined. We proposed and empirically tested a significant underlying mechanism, namely ego depletion. Thirdly, the role of social support is gaining prominence in helping employees cope with the challenges of COVID-19 telecommuting (Tu et al., 2021). This study explores the moderating effects of support from both supervisors and families, to identify effective resources to mitigate the potential adverse effects of supervisor control. The subsequent section elaborates on the relationships between research variables and formulates hypotheses.

## 2 Theory and hypotheses

### 2.1 The effect of supervisor control on employee outcomes

Telecommuting represents a new working arrangement, under which supervisor control may manifest in diverse forms and exert distinct effects on employees. Lautsch et al. (2009) compared management practices between telecommuting and non-telecommuting employees and discovered that, for telecommuting employees, close supervisor supervision could elevate work-family conflict and diminish performance. As some researchers found, if managers monitor behavior cannot be understood by employees, it is difficult for supervisor control to be effective (Felstead et al., 2003; Kurland and Cooper, 2002). While Wang et al. (2021) initially observed that monitoring could assist employees in overcoming procrastination in interviews, subsequent empirical research failed to establish its significant impact on performance. Instead, it revealed its adverse effects on work-family balance and wellbeing. Zheng et al. (2023) found that supervisor monitoring can make subordinates feel they are being “looked over their shoulders.” Such feelings are likely to reduce their sense of being trusted, and subsequently lead to exhaustion and diminished vigor on a given day. Taken together, prior studies have documented the complex and predominantly negative impacts of supervisor control for remote workers' work-family interface, psychological states, and job-related outcomes, yet limited research has explained the underlying mechanisms through which such control impairs employee effectiveness. Based on the JD-R model, we proposed that supervisor control could result in decreased task performance because, in such special times, supervisor control may impose an additional burden on individuals grappling with work and family demands. Additionally, supervisor control may be associated with heightened sleep problems due to employees must expend energy to meet their supervisor's demands.

**Hypothesis 1:** Supervisor control leads to low task performance (H1a) and high sleep problems (H1b).

### 2.2 The mediate effect of ego depletion

In the JD-R model, job demands initiate a health impairment process through exhaustion. This study employed ego depletion as an underlying mechanism in the health impairment process because it directly captures the sensation of one's energy depletion. According to ego depletion theory (Baumeister et al., 1998), individuals possess a finite pool of regulatory resources for self-control. Ego depletion transpires when these resources diminish due to regulatory demands. Supervisor control may be seen as a form of regulatory demand because supervisors need to supervise, maintain contact with, and elicit performance from telecommuting subordinates who are out of sight (Lautsch et al., 2009). Studies have shown that subordinates' burnout levels are linked to their perceptions of the leadership style exhibited by their direct supervisor. For example, Huang et al. (2020) discovered that employees regard abusive supervision as a kind of social and organizational job demand that intensifies emotional exhaustion, subsequently resulting in more frequent psychological withdrawal behaviors.

Furthermore, according to the JD-R model, one of the primary outcomes of exhaustion is poor health (Bakker and Demerouti, 2016), because exhaustion activates physiological systems that respond to stress and makes daily functioning more effortful (Headrick et al., 2022). Exhaustion has been demonstrated to be a contributing factor to sleep-related issues (Denollet and De Vries, 2006). Drawing on the self-regulation failure theory (Baumeister and Heatherton, 1996), ego depletion resulting from job demands has the potential to undermine individuals' self-regulation, subsequently resulting in poor sleep quality. For example, one study discovered that when employees work extended hours, their capacity to disengage from work is constrained, in turn, this process reduces sleep quality (Clinton et al., 2017). Besides, job demands could influence job performance via the induction of strain (Bilotta et al., 2021). De Clercq et al. (2022) found that abusive supervision increased employees' emotional exhaustion, further leading to their lower job performance. Thus, this study posited that supervisor control could lead to employee ego depletion, which further impacts both sleep quality and job performance.

**Hypothesis 2:** Ego depletion mediates the relationship between supervisor control and task performance (H2a), and sleep problems (H2b).

### 2.3 The moderate effect of support from supervisor and family

According to the “buffer” hypothesis of the JD-R model (Bakker et al., 2005), supervisor and family support could be regarded as social resources that mitigate the negative effects of supervisor control on employee ego depletion. While personal resources can account for individual differences in stress responses, social support appeared far more salient during the COVID-19 pandemic, given that people universally endured unavoidable social isolation that severely limited daily social contact and interpersonal interaction (Hodzic et al., 2024). Related studies have provided considerable evidence supporting the buffering effect of job resources in the connection between job demands and outcomes (e.g., Van den Tooren and de Jong, 2014). This suggests that when employees possess ample job resources to address their job demands, the adverse impacts of these demands are alleviated.

Several studies underscore the growing significance of social support within a remote workforce, which implies that individuals who lack social support may experience higher levels of emotional exhaustion because they cannot easily obtain instrumental or emotional support from their supervisors or colleagues (Vander Elst et al., 2017; Charalampous et al., 2021). Supervisor support encompasses the perception among employees that their supervisors foster autonomous goal setting, eliminate obstacles to goal attainment, and promptly offer constructive feedback and support (Rudolph et al., 2022). During the COVID-19 pandemic, research consistently highlights the critical buffering role of supervisor support. Chong et al. (2020) found that organizational support alleviated the link between employees' emotional exhaustion and withdrawal behavior. Hu et al. (2020) showed that servant leadership mitigated the negative impacts of state anxiety on work engagement and prosocial behavior. Hodzic et al. (2024) identified that without supervisor social support, telework led to reduced productivity, engagement, and increased social isolation via knowledge sharing.

In addition, family support represents another pivotal social support factor when working remotely, as it tends to be more accessible than support from supervisors or colleagues. Family support pertains to employees' perception of being valued, cared for, and esteemed by their families. This support enhances personal functioning and assists individuals in coping with stressors (Ersoy et al., 2023). During the COVID-19 crisis, Shin et al. (2021) identified a positive, long-term impact of family support on employee work-related outcomes. Additionally, Ersoy et al. (2023) revealed that both supervisor support and family support acted as moderators in the relationship between perceived uncertainty and emotional exhaustion. Family support operates through personal resource replenishment, making it a unique moderator by addressing the emotional and practical stressors of remote work that supervisor support may not fully cover. Thus, in the present study, both supervisor support and family support were viewed as playing critical roles in alleviating the impact of supervisor control on employee ego depletion. The following are the hypotheses of the moderation and moderation mediation effects.

**Hypothesis 3:** Supervisor support buffers the relationship between supervisor control and ego depletion (H3a), and supervisor support moderates the indirect relationship between supervisor control and task performance/sleep problems via ego depletion (H3b).

**Hypothesis 4:** Family support buffers the relationship between supervisor control and ego depletion (H4a), and family support moderates the indirect relationship between supervisor control and task performance/sleep problems via ego depletion (H4b).

**Figure 1:**
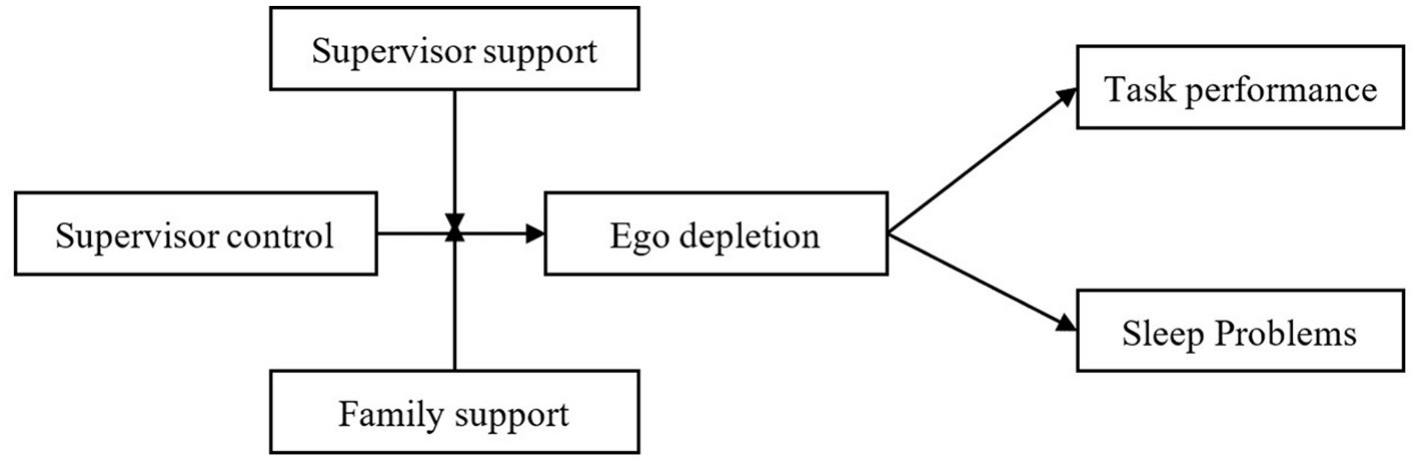
The conceptual model.

Figure 1 depicts the overall theoretical model.

## 3 Methodology

### 3.1 Sample and procedure

Participants were recruited that worked full-time remotely and had regular contact with their supervisors. Through snowball sampling, the data were collected in China during the nationwide lockdown under COVID-19. Survey data were collected at two time points separated by 2 weeks. In Time 1, data were collected at the end of February 2020, the fourth week of the nationwide lockdown. Participants completed online questionnaires to evaluate supervisor control and support behaviors, as well as provide demographic information. A total of 577 usable data were obtained at Time 1. In Time 2, questionnaires were administered to assess employees' ego depletion, sleep problems, and task performance. Data from Time 1 and Time 2 were matched using the last four digits of the participant's mobile phone numbers. A total of 447 responses were received at Time 2, and 303 of these could be matched with the first survey, resulting in a final effective response rate of 52.51%. Of the valid responses, 51.2% were males with an average age of 29.9 years old (*SD* = 7.64), 93.7% held a bachelor's degree or above, 57.4% were unmarried, and 26.1% had at least one child under 18 years old. The non-response bias test revealed no significant differences between the final sample and the missing sample in terms of gender, age, education level, and marital status, indicating the absence of non-response bias. The majority of the participants (69.0%) were general employees, 19.5% were first-line supervisors, and 11.5% were middle/senior supervisors. About half (44.9%) had never worked remotely before the COVID-19 pandemic, and about half (49.9%) had worked remotely occasionally. Only 5.3% had frequently worked remotely.

### 3.2 Measures

All scales were translated into Chinese using Brislin (1980) translation and back-translation procedure, based on well-established scales. Unless otherwise indicated, the items used a Likert-type scale anchored at 1 = strongly disagree to 5 = strongly agree.

Supervisor control and support behavior were measured with a 12-item developed by Gan et al. (2022). This scale includes two subscales: supervisor control (six items), and supervisor support (six items). Sample items include “During this COVID-19 work-from-home period, the supervisor pays too much attention to my work process” (supervisor control), and “During this COVID-19 work-from-home period, the supervisor provides me with the information and resources needed for work” (supervisor support). McDonald's ω was 0.87 and 0.84 for the supervisor control and support subscale.

Family support was measured with five items developed by King et al. (1995). These items were adapted for the telecommuting context with the prefix “When I work from home…” added. A sample item was: “When I work from home, my family tries not to disturb me.” McDonald's ω was 0.88 for this scale.

Ego depletion was measured with five items adapted from Twenge et al. (2004). A sample item was: “Right now, it would take a lot of effort for me to concentrate on something.” McDonald's ω was 0.85 for this scale.

Task performance was measured with six items developed by (Zhou (2005). A sample item was: “I always finish my work on time.” McDonald's ω was 0.93 for this scale.

Sleep problem was measured with four items from Jenkins et al. (1988). Participants were asked to evaluate “How often in the past month did you…,” an example item includes: “Have trouble falling asleep?” McDonald's ω was 0.82 for this scale.

We controlled for past telecommuting experience and the number of weeks of telecommuting because both are associated with employee work outcomes (Raghuram et al., 2001). We also controlled for age, gender, education, position at work, marital status, and having at least one child under 18: these demographic and family variables have been shown to shape teleworkers' work-related cognitions, home-work boundary demands and subsequent work outcomes (Gan et al., 2022).

### 3.3 Analysis strategies

First, we conducted a Confirmatory Factor Analysis (CFA) by Mplus 7.2 to determine the validity of our measures. Next, we used PROCESS v4.0 macro of SPSS 23.0 to test our hypotheses. PROCESS Model 9 was used to test the full hypothesis model. All indirect effects were calculated with 95% confidence intervals (95% CI) based on 5,000 bias-corrected bootstrapped samples.

## 4 Results

### 4.1 Preliminary analyses

As shown in Table 1, the CFA results suggested that our proposed six-factor model fit the data well [$\chi_{\left(449\right)}^{2}$ = 1,015.75, *p* < 0.001; CFI = 0.90, TLI = 0.89, and RMSEA = 0.06], and significantly better than all alternative models. In addition, we incorporated a common method factor into the six-factor model, constructing a seven-factor model. The results shown in Table 1 indicated an improvement in the fit; however, the enhancement in the CFI indicator is marginal, not exceeding 0.02, suggesting that common method variance does not pose a threat to the research findings. Harman's one-factor test was then used to test common method variance. The unrotated exploratory factor analysis results showed that the first factor accounted for 29.1% of the variance among variables, with 50% being the upper limit of acceptable explained variance on the first factor in Harman's test (Podsakoff et al., 2003). Thus, the model comparisons and Harman's one-factor test suggested that common method variance did not severely affect the results of our study.

**Table 1:** Confirmatory factor analysis results.

Model	Variables	χ^2^	df	χ^2^/df	RMSEA	CFI	TLI
Hypothesized model (Six-factor model)	SC; SS; FS; ED; TP; SP	1,015.75	449	2.26	0.06	0.90	0.89
Seven-factor model (include method factor)	SC; SS; FS; ED; TP; SP; method factor	917.72	418	2.20	0.06	0.91	0.89
Five-factor model	SC+SS; FS; ED; TP; SP	1,537.96	454	3.39	0.08	0.80	0.78
Four-factor model	SC+SS+FS; ED; TP; SP	2,164.34	458	4.73	0.11	0.69	0.66
Three-factor model	SC+SS+FS; ED; TP+SP	2,513.79	461	5.45	0.12	0.62	0.60
Two-factor model	SC+SS+FS; ED+TP+SP	3,128.75	463	6.76	0.13	0.51	0.48
One-factor model	SC+SS+FS+ED+TP+SP	4,843.27	464	10.44	0.17	0.20	0.14

*N*= 303.

SC, supervisor control; SS, supervisor support; FS, family support; ED, ego depletion; TP, Task performance; SP, sleep problem; RMSEA, root mean square error of approximation; CFI, comparative fit index; TLI, Tucker-Lewis index.

Means, standard deviations, and correlations appear in Table 2. The results showed that the correlation between supervisor control and supervisor/family support was non-significant; the correlation between supervisor control and ego depletion/sleep problem was positive and significant; the correlation between supervisor/family support and ego depletion was negative and significant, the correlation between support and task performance were positive and significant.

**Table 2:** Means, standard deviations, and correlations among study variables.

Variables	*M*	*SD*	1	2	3	4	5	6	7
1. Telecommuting weeks	3.12	1.35	–						
2. Telecommuting experience	1.82	0.90	0.16^**^	–					
3. Supervisor control	2.73	1.05	0.04	−0.06	–				
4. Supervisor support	3.60	0.76	0.13^*^	0.10	−0.01	–			
5. Family support	3.96	0.73	0.13^*^	0.03	−0.04	0.24^**^			
6. Ego depletion	2.71	0.96	−0.01	−0.02	0.25^**^	−0.14^*^	−0.12^*^	–	
7. Task performance	4.88	1.06	0.15^**^	0.04	0.06	0.28^**^	0.45^**^	−0.15^**^	–
8. Sleep problem	2.81	0.96	−0.01	0.05	0.26^**^	−0.03	0.02	0.22^**^	0.07

*N*= 303.

^*^p < 0.05, ^**^p < 0.01.

### 4.2 Hypothesis testing

In Table 3, supervisor control yielded a non-significant direct effect on task performance (*b* = 0.101, *p* = 0.087), suggesting that supervisor control had little substantive or practical influence on how well employees perform their tasks. Conversely, supervisor control demonstrated a moderate, statistically significant direct positive effect on health problems (*b* = 0.200, *p* < 0.001), indicating that higher levels of supervisor control were linked to more health problems. Thus, Hypothesis 1a was not supported while Hypothesis 1b was supported.

**Table 3:** The path estimates of the full model.

Outcomes	Ego depletion		Task performance		Sleep problem	
	*b*	*SE*	*b*	*SE*	*b*	*SE*
Control variables						
Gender	−0.161	0.109	−0.003	0.120	−0.093	0.108
Age	0.007	0.008	0.011	0.009	0.017^*^	0.008
Education	0.226^*^	0.105	0.243^*^	0.116	−0.040	0.104
Position	−0.039	0.072	0.236^**^	0.079	−0.052	0.071
Marriage	−0.017	0.121	−0.092	0.134	0.063	0.120
Child under 18	−0.027	0.146	0.096	0.161	0.010	0.144
Telecommuting weeks	0.004	0.041	0.091^*^	0.045	−0.020	0.040
Telecommuting experience	0.013	0.061	−0.005	0.067	0.069	0.060
Independent variables						
Supervisor control	0.211^***^	0.052	0.101	0.059	0.200^***^	0.052
Supervisor support	−0.177^*^	0.074				
Family support	−0.131	0.077				
Ego depletion			−0.203^**^	0.063	0.156^**^	0.057
Interaction term						
Supervisor control × Supervisor support	−0.131^*^	0.058				
Supervisor control × Family support	−0.030	0.072				
*R* ^2^	0.128^***^		0.114^***^		0.119^***^	
*F*	3.264		3.747		3.928	

*N*= 303.

^*^p < 0.05, ^**^p < 0.01, and ^***^p < 0.001.

The coefficients are unstandardized.

In Table 3, supervisor control had a small-to-moderate and significant positive effect on ego depletion (*b* = 0.211, *p* < 0.001), while ego depletion had a small but significant negative effect on task performance (*b* = −0.203, *p* < 0.01), and had a small but significant positive effect on sleep problem (*b* = 0.156, *p* < 0.01). The bootstrap test results revealed a small but significant negative indirect effect of supervisor control on task performance (indirect effect = −0.041, 95% CI = [−0.082, −0.010]), and a small but significant positive indirect effect on sleep problems (indirect effect = 0.031, 95% CI = [0.006, 0.065]). Although these effect sizes appear small, their practical significance should not be overlooked: even modest levels of supervisor control can gradually erode self-regulatory resources, impair daily task execution, and disrupt sleep over repeated daily working episodes. Overall, Hypothesis 2 was supported.

**Figure 2:**
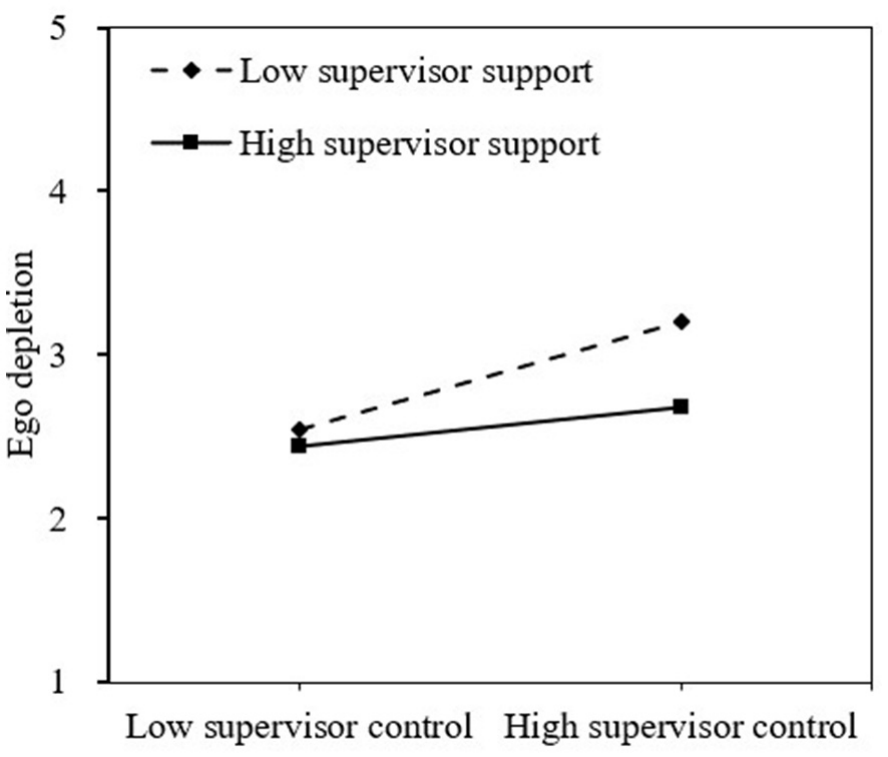
The interactive effect of supervisor control and support on ego depletion.

The results in Table 3 demonstrated that the interaction between supervisor control and support for ego depletion was significant (*b* = −0.131, *p* < 0.05). To elucidate the moderating role of supervisor support, we plotted the relationship between supervisor control and ego depletion at 1 *SD* above and below the mean of supervisor support. As illustrated in Figure 2, when supervisor support was low, the relationship between supervisor control and ego depletion was significant (*b* = 0.288, *t* = 4.962, *p* < 0.001, and 95% CI = [0.174, 0.403]). However, when supervisor support was high, the relationship became non-significant (*b* = 0.113, *t* = 1.581, *p* = 0.115, and 95% CI = [−0.028, 0.254]). Therefore, these results offered support for Hypothesis 3a. However, involving the moderating role of family support, the interaction between supervisor control and family support regarding ego depletion yielded non-significant results (*b* = −0.030, *p* = 0.676), thus not supporting Hypothesis 4.

To test the moderated mediation models, Edwards and Lambert (2007) moderated path analysis approach was applied. Regarding the indirect effect of supervisor control on task performance, when supervisor support was above 1 *SD*, the indirect effect was non-significant (indirect effect = −0.023, 95% CI = [−0.068, 0.005]), when supervisor support was below 1 *SD*, the indirect effect was significantly negative (indirect effect = −0.064, 95% CI = [−0.116, −0.020]), and the difference between two conditions was significant (contrast effect = 0.041, 95% CI = [0.000, 0.085]). Concerning the indirect effects of supervisor control on sleep problems, when supervisor support was above 1 *SD*, the indirect effect was non-significant (indirect effect = 0.017, 95% CI = [−0.004, 0.051]), when supervisor support was below 1 *SD*, the indirect effect was significantly positive (indirect effect = 0.049, 95% CI = [0.010, 0.097]), but the difference between two conditions was nonsignificant (contrast effect = −0.032, 95% CI = [−0.076, 0.001]). Overall, Hypothesis 3b received partial support.

## 5 Discussion

Based on the JD-R model, this study investigated the impact of supervisor control on employee performance and wellbeing during the COVID-19 telecommuting period. The research findings provide support for the majority of our hypotheses. We identified ego depletion as a mediator in the link between supervisor control and both employee task performance and sleep problems. Furthermore, our research revealed a buffering effect of supervisor support on the relationship between supervisor control and ego depletion. Theoretical and practical implications are outlined below.

### 5.1 Theoretical implications

This study made three contributions to the existing literature. First, within the context of COVID-19 telecommuting, we directed our attention to employees' performance and wellbeing outcomes associated with supervisor control. The performance and health of employees are identified as key indicators for telecommuting success, as telecommuter job performance is a matter of ongoing public debate, and telecommuting carries both positive and negative effects on workers' physical and mental health (Vacchiano et al., 2024). In remote working arrangements, supervisors and subordinates are no longer in the same physical space, which often leads supervisors to perceive monitoring as even more important (Zheng et al., 2023). The behaviors supervisors exhibit in managing subordinates can profoundly impact employee performance and wellbeing. Our research conceptualizes supervisor control as a distinct job demand, thereby enriching the categories of demands within the JD-R model and validating the model's applicability in explaining the impact of leadership behaviors in the context of telecommuting.

Second, based on the JD-R model, we expand upon existing knowledge regarding the impact of supervisor control on employees' outcomes. Consistent with the JD-R model, we observed that ego depletion may act as a mediating mechanism. Specifically, supervisor control appears to induce employees' ego depletion, subsequently reducing their task performance and increasing sleep problems; this finding aligns with prior research (e.g., Denollet and De Vries, 2006; Sardeshmukh et al., 2012; De Clercq et al., 2022). Ego depletion represents a feeling of energy-draining and may be caused by excessive demands, ultimately leading to health impairment. For teleworkers, supervisor control can be seen as an additional demand, because it arises from special work patterns and consumes employees' self-regulation resources on somewhat unnecessary tasks, such as clocking in and out via computer applications. This supervision may cause employees to feel a lack of trust in their organization (Parker et al., 2020; Zheng et al., 2023), a scarcity of self-regulation resources can exacerbate harm to employees' work performance (De Clercq et al., 2022) and hinder their ability to recover from work-related stressors (Clinton et al., 2017). Notably, supervisor control exerted no direct significant effect on task performance. This pattern may arise because supervisor control simultaneously exerts both positive and negative effects on work performance (Wang et al., 2021; Kniffin et al., 2021), and these opposing effects offset each other, resulting in a non-significant direct relationship. Our research reveals that ego depletion serves as a key mechanism for explaining the negative effects of supervisor control.

Third, our study revealed that supervisor support can buffer the negative effect of supervisor control on ego depletion, consistent with prior research (e.g., Van den Tooren and de Jong, 2014). Leader factors have consistently been regarded as vital social resources that assist employees in overcoming the uncertainty brought on by the pandemic (Ersoy et al., 2023). Telecommuters who received increased social support from supervisors reported experiencing less social isolation and reduced psychological strain (Bentley et al., 2016). Furthermore, prior studies did not yield a consensus regarding the relationship between supervisor control and employee performance. Our findings showed that only when supervisor support was low did supervisor control lead to ego depletion and low task performance. Therefore, we provide an explanation perspective for clarifying previous unstable results. Unexpectedly, we did not find the moderation effect of family support, contradicting the findings of Tu et al. (2021) and Ersoy et al. (2023), both of which suggested that family support could mitigate the association between COVID-19 uncertainty and perceived stress. This result can be explained from two aspects. On the one hand, supervisor control is not a COVID-19 shock event that needs sufficient attention from the family. On the other hand, the support from family may not primarily target the alleviation of work-related stressors; and compared with supervisor support, the spillover effect of family support on work-related variables may be weaker.

### 5.2 Practical implications

Our research offers two recommendations for management practices. First, as Jeske (2022) summarized, managers should heighten their awareness of how their leadership style, performance monitoring, and feedback can directly or indirectly (via their use of certain tools) influence employee performance and wellbeing over time (Spagnoli et al., 2020; Wang et al., 2021). Our study revealed that supervisor control may initiate a health impairment process. Prior research has suggested that limitations in supervisors' decision-making authority or excessive administrative duties can impede the success of telecommuting (Bloom et al., 2015; Brodt and Verburg, 2007). Hence, we recommend that supervisors should enhance their trust in telecommuting employees and reduce the intensity of performance monitoring (Greer and Payne, 2014), such as replacing frequent clock-ins with weekly outcome-based check-ins and eliminating redundant metrics. Meanwhile, it's important to note that supervisor control is directly tied to the organization's bottom line and the survival of the firm in tough circumstances (Allen et al., 2015). Thus, supervisors can set clear, mutually agreed work arrangements through flexibility idiosyncratic deals (I-deals), delegate appropriate decision-making authority, thereby transforming monitoring pressure into motivation.

Second, our study identified that supervisor support can mitigate the detrimental effects of supervisor control on ego depletion. As Gohoungodji et al. (2022) highlighted, organizational support stands as a pivotal success factor for telecommuting. Receiving social support while working remotely can assist individuals in overcoming social isolation (Bentley et al., 2016). To provide effective support, leaders can improve their management skills and capabilities through continuous practice and learning. For instance, Jackson (2022) suggested that organizational leaders and HRD practitioners should apply the experiential learning theory to guide their learning from experiences in dealing with remote work challenges. Supervisors can develop new approaches attuned to the needs of employees in new flexible arrangements (i.e., increased information sharing and assistance in boundary management; Lautsch et al., 2009), and build trust within the distributed team (Grant et al., 2013). Furthermore, digital monitoring systems embody broader patterns of technology acceptance, which is determined by perceived usefulness and perceived ease of use (Poorirerngpoom et al., 2025). Leaders can boost employee acceptance of such digital tools through targeted technical support, such as offering step-by-step operation guidance, simplifying tool functions, and setting up timely online help services.

### 5.3 Limitations and directions for future research

Several limitations of the study need to be acknowledged. First, all of our data were collected through self-report, which is inevitably affected by common method variance; given that we collected data at two-time points to mitigate this issue (Podsakoff et al., 2003). Future research may consider longitudinal designs or utilize a combination of self-reported and other-reported data, such as supervisor-rated performance.

Second, merely treating supervisor control as a job demand is oversimplified; prior research suggests it can also have motivational or performance-enhancing effects under certain conditions (e.g., Wang et al., 2021). This dual nature of supervisor control may explain the non-significant results regarding the direct relationship between supervisor control and employee performance in our study. Additionally, different levels of supervisor control (e.g., high control increasing stress; Kniffin et al., 2021) and its dual effects via distinct mechanisms further underscore this complexity. Future research should integrate supervisor control's dual nature, explore curvilinear effects and alternative mechanisms, to clarify inconsistencies in prior findings.

Third, in our study, the moderation effect of family support was not found. Prior research has demonstrated that family support can produce a positive spillover effect on work-related variables. For example, Shin et al. (2021) found that pre-COVID-19 family support can bring mid-COVID-19 high job performance and organizational citizenship behavior through a reduction in mid-COVID-19 emotional exhaustion. Further research should provide more evidence about the mechanisms by which family support functions in addressing work-related demands.

## 6 Conclusion

This study extends telecommuting and COVID-19 literature by investigating the relationship between supervisor control and employees' task performance and sleep problems, with a particular focus on ego depletion as the mediating factor based on the JD-R model. This study underscores the significance of supervisor support in mitigating the adverse effects of supervisor control on ego depletion, in turn, could not undermine task performance. Besides, the role of family support needs further exploration. We hope this research inspires further investigation of how and when supervisor behaviors can contribute positively to large-scale telecommuting.

## Data Availability

The original contributions presented in the study are included in the article/supplementary material, further inquiries can be directed to the corresponding author/s.
